# Glycaemia in low-premixed insulin analogue type 2 diabetes patients in a real-world setting: are the CGM targets met?

**DOI:** 10.1186/s40001-023-01081-y

**Published:** 2023-03-07

**Authors:** Mitja Krajnc, Nika Aleksandra Kravos Tramšek

**Affiliations:** Department of Endocrinology and Diabetology, University Medical Center Maribor, Maribor, Slovenia

**Keywords:** Type 2 diabetes (mellitus), Premixed insulin (therapy), Continuous glucose monitoring (CGM), CGM targets, Real-world study

## Abstract

**Background:**

There are insufficient data on continuous glucose monitoring (CGM) in nonintensive insulin therapy patients. Using CGM and the recommended CGM targets, we wanted to evaluate low-premix insulin analogue therapy (biphasic aspart/NovoMix 30 and biphasic lispro 25/Humalog Mix 25) in real-world type 2 diabetes patients for glycaemic efficacy and especially hypoglycaemia.

**Methods:**

The prospective observational study was performed on 35 patients who were treated with a low-premixed insulin. We used the Dexcom G6 system for CGM (9.6 ± 1 days) to measure the clinically relevant CGM parameters: glycaemic variability (%CV), TBR (time below range) < 3.0 mmol/l = 54 mg/dl (level 2 hypoglycaemia), TBR 3.0–3.8 (= 54–69 mg/dl), TIR (time in range) 3.9–10–0 mmol/l (70–180 mg/dl), TAR (time above range) 10–13.9 mmol/l (180–250 mg/dl) and TAR > 13.9 mmol/l (250 mg/dl). We also assessed clinical and demographic characteristics, laboratory HbA1c, fasting blood glucose, peak postprandial glucose values, and the percentage of hypoglycaemia between 00:00 and 06:00.

**Results:**

In our patients, the average ± SD age was 70.4 ± 9.2 years, diabetes duration 17.4 ± 7.1 years, 51% were females, average daily insulin dose was 46.4 units (80% received biphasic aspart). The average ± SD TIR was 62.1 ± 12.2%, TBR < 3.0 mmol/l 0.8 ± 2.0%, TBR 3.0–3.8 mmol/l 1.5 ± 1.5%, TAR 10–13.9 mmol/l 29.2 ± 12.4%, TAR > 13.9 mmol/l 6.4 ± 7.2% and %CV 29.9 ± 7.1%. The average time in hypoglycemia was 33.1 min daily in our patients (11.5 min in the level 2 range). In the older/high-risk population, the TBR/TIR/TAR/level 2 TAR targets were met in 40/80/77/80%, respectively. For the general T2D people, level 2 TBR/TBR/TIR/TAR/level 2 TAR would be met in 74/83/34/77/49%. Average fasting blood glucose was 8.0 ± 2.5 mmol/l (144 ± 45 mg/dl), BMI 31.3 ± 5.1 kg/m^2^, daily insulin dose 46.4 ± 12.1 units, HbA1c 57.4 ± 5.4 mmol/mol (7.4 ± 0.7%). The glycaemic variability goal was met in 80% (with 66% meeting the lower 33% CV goal). 17 ± 12% of hypoglycaemia was nocturnal. People with TBR > 4% were significantly older.

**Conclusions:**

Most of our type 2 diabetes patients, treated with low-premixed insulin, did not meet the recommended TBR target for older/high-risk patients while meeting the TIR and TAR targets. Nevertheless, the time spent in (total and nocturnal) hypoglycemia was short. The study indicates that the general type 2 diabetes population targets would mostly be met for TBR and %CV in our patients but not the TIR and TAR targets. CGM appears to be a useful clinical tool in these patients.

## Introduction

From 2018 to 2030, the world insulin requirements are expected to increase parallel to the incidence of diabetes by more than 20% [1]. In modern patient-centred and individualised type 2 diabetes (T2D) management, it is necessary to address the adverse effects of medications, key person characteristics, the complexity of regimens (including frequency), access, cost and availability of drugs [2, 3]. The most significant reduction in HbA1c is seen with insulin regimens and GLP1 receptor agonists [4]. Insulins do not offer additional cardiovascular or renal benefits, cause weight gain and require subcutaneous injections. The currently recommended way of starting insulin therapy in people with T2D is the addition of basal insulin to the prior pharmacological treatment in conjunction with revisiting health behaviour and diabetes self-management education and support. Only when combination therapies with basal insulin are no longer sufficient, the next step is to further intensify therapy with prandial insulin [2, 3, 5].

Historically, premixed insulins have frequently been prescribed to persons with T2D. 2012 ADA and EASD recommendations stated that the most convenient strategy for insulin initiation was a single basal insulin injection, and the most precise and flexible prandial coverage was possible with basal-bolus therapy. Premixed insulin, consisting of a fixed combination of intermediate insulin with a rapid analogue or the regular insulin, was considered somewhat inflexible but appropriate for certain patients who ate regularly and needed a simplified approach beyond basal insulin. In general, when compared with basal insulin alone, premixed regimens tend to lower HbA1c to a more significant degree, but often at the expense of more hypoglycemia and weight gain. Disadvantages include the inability to titrate the shorter from the longer acting component of the prescribed formulations [6, 7]. There is less weight gain, less hypoglycemia and lower insulin dose with premixed insulin analogues compared to basal-bolus therapy [3, 8]. Still, the choice of an insulin regimen for initiation or intensification of therapy is a subjective decision, that should consider the duration of diabetes, symptoms of hyperglycemia, lifestyle, drug therapy, glycaemic status (with patterns of glycaemia, risk of hypoglycemia and glycaemic variability) and patient preference [9]. Premixed insulin is sometimes considered the best option in patients who are unwilling or unable to adhere to the increased number of injections and monitoring required with basal plus/basal-bolus regimens [10]. Two doses of premixed insulin are a simple, convenient means of spreading insulin across the day and are usually considered when basal or basal-plus insulin is no longer sufficient to reach an individual's A1c target [3]. Despite national and international guidelines, the proportions of insulin regimens differ substantially between European countries. Premixed insulins are still widely used in some countries [11]. E. g. premix use, already high as a starting regimen, was used by one-third of the participants after 4 years in Northern Europe in CREDIT non-interventional study, with authors suggesting a lower number of injections as a probable cause [12].

In our study, we wanted to evaluate low-premix analogue therapy (biphasic insulin aspart, containing 30% soluble insulin aspart and 70% protamine-crystallized insulin aspart/NovoMix 30 and biphasic lispro 25, containing 25% soluble insulin lispro and 75% protamine-crystallized insulin lispro/Humalog Mix 25) for glycaemic efficacy and the occurrence of hypoglycaemia. We performed the study in a real-world setting, using a continuous glucose monitoring (CGM) system. The results would help us consider the potential need for a change in selected antihyperglycaemic therapy. Following the guidance [2, 3, 13], combination therapy including a basal insulin, basal-bolus regimen and fixed basal insulin/GLP1 receptor antagonist combination could all be considered the antihyperglycaemic alternatives of premixed insulin.

Numerous studies have demonstrated significant clinical benefits of CGM use regardless of insulin delivery method, also for people with T2D [14, 15]. Nevertheless, CGM data in patients treated with premixed insulin are scarce, and the evidence is insufficient for people with a nonintensive insulin regimen [3, 16]. The primary CGM characteristic of effective and safe glucose control is high time in range (TIR) and low time below range (TBR), in particular for blood glucose (BG) below 3.0 mmol/l (54 mg/dl). The international guidance on targets for assessment of glycemic control for the general adult population with T2D recommends aiming for > 70% of readings in TIR 3.9–10.0 mmol/l (70–180 mg/dl), < 4% of readings below 3.9 mmol/l; < 1% of readings below 3.0 mmol/l (54 mg/dl, level 2 hypoglycaemia), < 25% of readings above 10.0 mmol/l (180 mg/dl) and < 5% of readings above 13.9 mmol/l (250 mg/dl, level 2 hyperglycaemia). For older and high-risk people with T2D, the targets are less stringent in the interest of safety: > 50% of readings should be in TIR, less than 1% below 3.9 mmol/l and less than 50% above 10.0 mmol/l (with less than 10% above 13.9 mmol/l). Evidence regarding CGM for this group is lacking but the elevated risk of hypoglycemia has been well-documented. The goal for glycaemic variability (coefficient of variation) should be ≤ 36% [14]. Hypoglycemia is especially a threat during the night in patients with insulin-treated T2D, which may lead to increased mortality, anxiety, poor adherence, and hypoglycemia unawareness [17]. Nocturnal hypoglycemia is usually defined by a time window for which the international consensus recommendation is from midnight to 6 am, which generally includes the duration of nighttime sleep and the longest inter-prandial interval [18].

While CGM provides insights into the daily glucose fluctuations, it can also be used to calculate an estimated HbA1c and expressed as glucose management indicator (GMI) [19]. Clinical studies are an excellent application for intermittent CGM, and CGM parameters are nowadays regarded as mandatory for documenting clinical trials [20]. Through a broad spectrum of glucose-derived data, CGM is a valuable tool for clinically evaluating glucose-lowering medications [21].

## Methods

We conducted the prospective observational study on 35 persons with T2D treated in our institution's diabetology outpatient clinic (Maribor University Medical Centre provides secondary and tertiary medical care for northeastern Slovenia; Slovenian insulin-treated patients are not usually followed-up in primary care). Our participants were randomly selected from all premixed insulin patients from June to December 2022. Simple random sampling was performed by a computer programme that generated a random number, corresponding to a patient file number. All the patients regularly received diabetes self-management education and support (with a more extended structured programme at an insulin initiation and then periodically at follow-up visits and when requested by a patient or a healthcare professional). Our institution's medical ethics committee approved the study in October 2021. We calculated the sample size to estimate a simple mean for TBR < 3.0 mmol/l (54 mg/dl) and TBR 3.0–3.8 mmol/l (54–69 mg/dl) to be at least 25, with the assumptions of standard deviation 1.0 (based on pilot data) and required size of standard error 0.2.

The inclusion criteria were the following: signed written informed consent after the thorough familiarisation with the study, age above 18 years, T2D diagnosed for at least 6 months, current treatment with a premixed insulin analogue preparation: BiAsp 30/biphasic aspart 30 (NovoMix 30 by Novo Nordisk) or biphasic lispro mix 25 (Humalog Mix 25 by Eli Lilly). The exclusion criteria were HbA1c above 85.8 mmol/mol (10.0%), current use of a concomitant medication that could affect glycaemia or sensor accuracy (e.g., a systemic glucocorticoid, hydroxyurea, high doses of paracetamol), end-stage renal disease, pregnancy and breastfeeding. One-quarter of eligible patients refused to participate in the study.

In our participants, we collected data on the following parameters: age, sex, T2D duration, daily insulin dose and number of daily injections, concomitant use of metformin, an SGLT2 inhibitor or an incretin-based medication, fasting BG and HbA1c before the sensor insertion, body weight and body mass index and a known diagnosis of at least one diabetic microvascular and/or at least one macrovascular complication. Fasting BG and IFCC-standardized HbA1c were measured in our institution's central laboratory within 10 days before a sensor insertion.

For continuous glucose monitoring, we used a real-time Dexcom G6 personal system (Dexcom, Inc., San Diego, California). It does not require calibration and can be worn for up to 10 days, which was also our goal. Its overall MARD (mean absolute relative difference) is 9.0% [20, 21]. We used devices in a blinded fashion, with the participants educated on the proper conduct for interstitial glucose data collection via a receiver. The participants were also instructed to lead their usual daily routine and to continue with their current diabetes and other treatments. A Dexcom G6 sensor was inserted on day 1 by an experienced nurse educator with an auto-applicator, and the participants were instructed on good sensor care. A sensor was removed after 10 days (or earlier if there were technical difficulties or a participant requested it). The specialized Dexcom Clarity software was used. From the CGM data, we recorded the percentage of TIR, TBR (3.0–3.8 mmol/l, 54–70 mg/dl), TBR (< 3.0 mmol/l, 54 mg/dl), time above range (TAR) (10–13.9 mmol/l, 180–250 mg/dl), TAR (> 13.9 mmol/l, 250 mg/dl), glycaemic variability % (CV%) and glucose management indicator (GMI). For each participant, we also visually determined average peak postprandial BG after breakfast, lunch and dinner (within 3 h postprandial period) from the Dexcom Clarity CGM graph, based on the participants' self-reported times of the individual main meals. From the Dexcom Clarity analysis, we also recorded the percentage of total TBR occurring during the nocturnal period (between 00:00 and 06:00; nocturnal hypoglycemia).

We performed statistical analysis with SPSS Statistics, version 29.0.0.0 (Chicago, IL, USA). We analysed data to acquire averages, standard deviations, minimum and maximum values of the chosen parameters, and the frequencies stratified by clinically relevant intervals for a selected variable. We performed Wilcoxon rank sum test to assess whether the examined parameters differed significantly between the two TBR groups (TBR < 4% vs. ≥ 4%). *P* value of < 0.05 was considered to be statistically significant.

## Results

The study included 35 participants. Clinical and demographic characteristics are shown in Table 1. The mean age of the participants was 70.4 years, 51% were women, and all were white; the mean diabetes duration was 17.4 years and the insulin treatment duration was 7.6 years. The average laboratory serum fasting glucose was 8.0 mmol/l (144 mg/dl) and the average HbA1c was 57.4 mmol/mol (7.4%). The average total daily insulin dose was 46.4 units in 2.2 daily doses. In average, we acquired 9.6 days of sensor data in a participant (that is 336 patients-days in all the participants). 80% of the participants were treated with biphasic aspart, the rest with biphasic lispro. Considering concomitant medications, 69% were also treated with metformin, 31% with a SGLT2 inhibitor, 17% with a DPP-4 inhibitor, 6% with a GLP-1 receptor agonist and none with a sulphonylurea or a glinide. 69% of patients had at least one microvascular complication and 37% had at least one macrovascular complication of diabetes. The mean BMI was 31.3 and the mean relative body change in the previous 5 years was + 3.6%.

**Table 1:** Clinical and demographic characteristics of our patients (N = 35)

	Minimum	Maximum	Mean	Std. deviation
Age (years)	52	90	70.4	9.2
Diabetes duration (years)	5	28	17.4	7.1
Insulin treatment duration (years)	2	19	7.6	3.4
Fasting BG (mmol/l (mg/dl))	2.4 (43.2)	16.7 (300.6)	8.0 (144)	2.5 (45)
HbA1c (mmol/mol (%))	44.3 (6.2)	74.9 (9.0)	57.4 (7.4)	5.4 (0.7)
Body weight (kg)	61	115	84.6	14.4
Body mass index (kg/m2)	24	46	31.3	5.1
Waist circumference (cm)	85	132	110	12
Daily total insulin dose (units)	24	78	46.4	12.1
Daily insulin doses (number/day)	2	3	2.2	0.4
CGM active sensor data (days)	6	11	9.6	1
Relative body weight change in the previous 5 years (%)	− 6	8	1.8	3.6

	*N*	%
Female sex	18	51
Premixed insulin	Aspart 30: 28 Lispro 25: 7	80 20
Metformin therapy	24	69
SGLT2 inhibitor therapy	11	31
DPP-4 inhibitor therapy	6	17
GLP-1 receptor agonist therapy	2	6
Insulin secretagogues therapy	0	0
HbA1c (mmol/mol (%))	To 53 (7.0): 9 53 (7.0)–69.4 (8.5): 19 69.4 (8.5) + : 7	26 54 20
At least 1 microvascular complication of diabetes	24	69
At least one macrovascular complication of diabetes	13	37

*CGM* continuous glucose monitoring, *BG* blood glucose, *N* number

In our participants, the average TIR was 62.1%, TBR 2.3% and TAR 35.6%, with 0.8% of time below 3.0 mmol/l and 6.4% of time above 13.9 mmol/l. Average %CV was 29.9. Mean laboratory HbA1c was equal to the calculated glucose management indicator (7.4). The average glucose after lunch and dinner was 12.0 mmol/l (216 mg/dl). On average, 17% of hypoglycaemia occurred in the period between 00:00 and 06:00. Table 2 presents our data in standardised CGM metrics (based on 14). Data on average peak postprandial glucose after three main meals and the percentage of total TBR during 00:00–06:00 period (nocturnal hypoglycaemia) are also included.

**Table 2:** Glycaemic data in standardised CGM metrics, average peak postprandial BG and percentage of nocturnal TBR

	Minimum	Maximum	Mean	Std. deviation
TIR, time in range 3.9–10.0 mmol/l (70–180 mg/dl) (%)	42	89	62.1	12.2
TBR, time below range 3.0–3.8 mmol/l (54–70 mg/dl) (%)	0	5.2	1.5	1.5
TBR, time below range < 3.0 mmol/l (54 mg/dl) (%)	0	10.4	0.8	2.0
TAR, time above range 10.0–13.9 mmol/l (180–250 mg/dl) (%)	11.0	58.2	29.2	12.4
TAR, time above range > 13.9 mmol/l (250 mg/dl) (%)	0	28.0	6.4	7.2
glycaemic variability (%CV)	15.8	47.7	29.9	7.1
glucose management indicator (GMI)	6.2	8.7	7.4	0.7
BG after breakfast (mmol/l (mg/dl))	8.0 (144)	18.0 (324)	11.2 (201.6)	2.0 (36)
BG after lunch (mmol/l (mg/dl))	3.0 (54)	19.0 (342)	12.0 (216)	2.8 (50.4)
BG after dinner (mmol/l (mg/dl))	9.0 (162)	18.0 (324)	12.0 (216)	2.5 (45)
Percentage of total TBR during 00:00–06:00 time period (nocturnal hypoglycaemia)	0	36	17	12

*BG* blood glucose, *%CV* coefficient of variation in %

34% of the participants achieved the recommended general T2D glycaemic goals [14] for TIR, 83% for TBR below 3.9 mmol/l (74% for values below 3.0 mmol/l) and 23% for TAR (51% for values above 13.9 mmol/l). 83% of the participants achieved older/high-risk population goals for TIR, 40% for TBR, and 77% for TAR (80% for values > 13.9 mmol/l). 80% of the participants met the general %CV goal (of ≤ 36%). In Table 3, we present the stratified frequencies for selected glycaemic parameters.

**Table 3:** Stratified frequencies for selected glycaemic parameters

	*N*	%
TIR, time in range (3.9–10.0 mmol/l) (%)	< 50%: 6 50–70%: 17 > 70%: 12	17 49 34
TBR, time below 3.9 mmol/l (70.2 mg/dl) (%)	< 1%: 14 < 4%: 29 ≥4%: 6	40 83 17
TBR, time < 3.0 mmol/l (54 mg/dl) (%)	< 1%: 26 1–3%: 8 > 3%: 1	74 23 3
TAR, time above 10.0 mmol/l (180 mg/dl) (%)	< 25%: 8 25–50%: 19 > 50%: 8	23 54 23
TAR, > 13.9 mmol/l (250 mg/dl) (%)	< 5%: 18 5–10%: 10 > 10%:7	51 29 20
glycaemic variability (%CV)	≤ 36%: 28 > 36%: 7 < 33%: 23	80 20 66
percentage of TBR during the period 00:00–06:00	0%: 22 1–10%: 6 11–30%: 5 30+%: 2	63 17 14 6

% *CV* coefficient of variation in %, *TIR* time in range, *TBR* time below range, *TAR* time above range

**Fig. 1:**
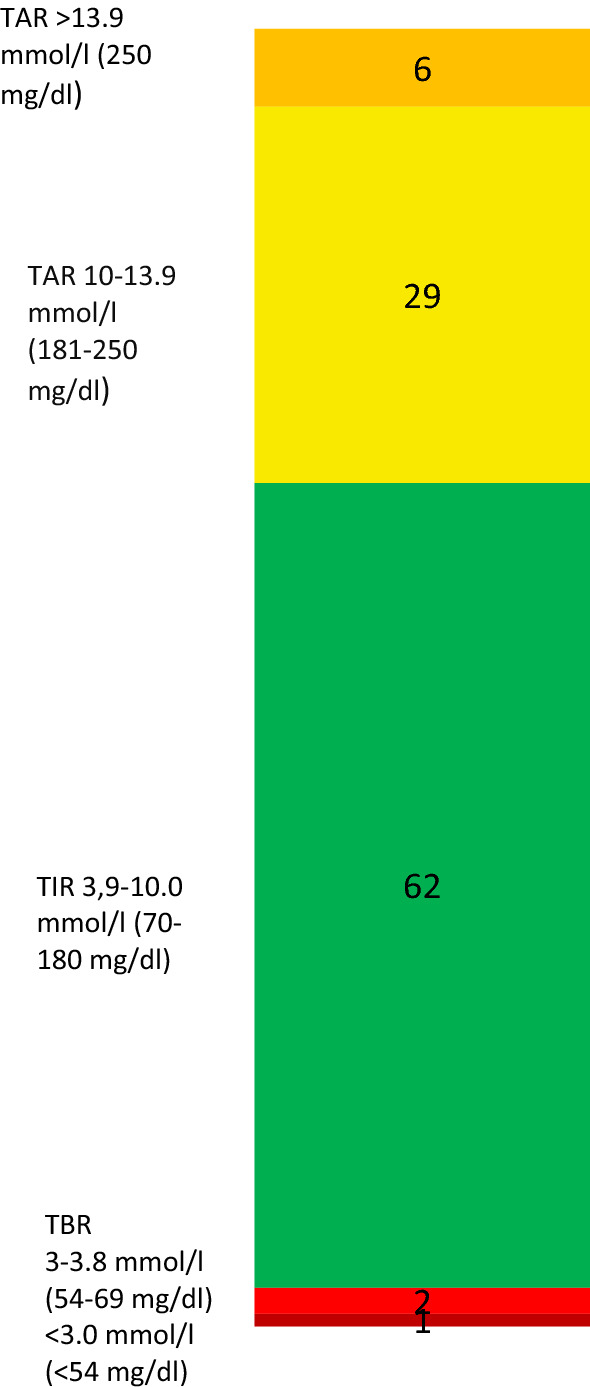
The average CGM metrics of our participants. Times are presented as %. TAR: time above range (orange: >13.9 mmol/l, yellow: 10-13.9 mmol/l), TIR: time in range (green), TBR: time below range (light red: 3-3.8 mmol/l, dark red: <3.0 mmol/l)

We present the average CGM metrics of our participants in Fig. 1.

We performed Wilcoxon rank sum test to determine whether the two TBR groups (TBR < 4% vs. ≥ 4%) differed by age, sex, T2D duration, daily insulin dose, fasting BG, HbA1c, body weight or body mass index. Based on the sum of ranks for TBR ≥ 4% (*N* = 9), *p* value of < 0.05 was found for age (*T* value 162). The differences in other parameters were insignificant (*p* > 0.05).

Twelve eligible and invited patients (26%) refused to take part in the study. They tended to be older, male and with longer diabetes duration.

## Discussion

In our T2D real-world patients treated with a low-premixed insulin preparation, the average time spent in hypoglycemia was 33.1 min daily (2.3%), with 11.5 min in level 2 hypoglycemia. In the general type 2 diabetes population, that would translate to reaching the CGM TBR targets [14] in the majority of patients: 74% for level 2 and 83% for all hypoglycemia. Our patients, however, were older on average [24–26] and at higher risk for hypoglycemia, which is more common in the elderly, with longer duration of diabetes and in insulin-treated people [3, 28]. In addition, in our population, people with TBR ≥ 4% were significantly older than people with TBR < 4% but did not differ in the other studied parameters. Considering the safer hypoglycaemia-preventing guidance for this group [14], only 40% of patients would reach the more strict TBR target of less than 1% below 3.9 mmol/l. Nevertheless, the average TBR was quite short in our study. Wang et al. measured average TBR < 3.9 mmol/l of 9.4% for a premixed insulin group (*n* = 194) [26], and Margaritidis et al. the value of 6.3% in a well-controlled group (*n* = 36) [27]. Lin et al. reported a TBR value of 0 for their 16 premix patients [29].

In our study, 17% of hypoglycaemia occurred during the most vulnerable nocturnal period (between 00:00 and 06:00), which is less than is expected in higher risk patients, e.g., type 1 or advanced insulin-dependent type 2 diabetes patients [17, 18]. 63% of the participants did not experience nocturnal hypoglycaemia at all. The lower risk of nocturnal hypoglycaemia in the participants could be linked to a ratio of prandial/basal insulin, residual beta cell function and insulin secretion, the more pronounced tendency to hyperglycaemia in premix patients and behavioural modification. There are emerging data that some CGM parameters could help prevent nocturnal hypoglycaemia in insulin-treated T2D patients [17].

In contrast, the TIR targets for high-risk T2D people [14] were mostly (in 80%) met in our patients, with only 34% meeting the general population target. Wang et al. showed a similar TIR of 59.8% for their group [26]. Lin et al. reported a TIR of 39.0% for premix-treated patients [29], and Margaritidis et al. measured a value of 74.2% [27]. The TAR target for high-risk people was met in 77% of our group. 77% of patients did not meet the more stringent general population hyperglycaemia goal, with 49% not satisfying the level 2 hyperglycaemia goal. Wang et al. also reported level 2 hyperglycemia 3.8% of the time [26]. In the study by Margaritidis et al., TAR was 19.6% in a well-controlled group [27]. Lin et al. reported a TAR of 61.0% [29]. The general glycaemic variability target was met in 80% of our patients. Some studies suggest that the lower %CV value of < 33% provides additional protection against hypoglycaemia in patients receiving insulin [14, 30], which was reached by 66% of our patients. Wang et al. report a higher average %CV of 36.1% for premix patients [26]; in the study by Margaritidis, it was 34.2% [27]. In the study by Lin et al., it was 24% [29].

There is a growing body of evidence suggesting that CGM use confers benefits in individuals who are treated with less-intensive therapies, among them better glycaemic control (e.g., lower HbA1c), reduced resource utilisation and costs, and also (beyond diabetes) CGM-based behavioural interventions and quality of life [31]. Our results indicate that CGM is clinically useful in individual premix-treated patients to reveal hypo- and hyperglycaemic patterns and time outside the range. That could be especially important in older/high-risk people with multiple hypoglycemic risk factors. As always, glycaemic targets should be individualised, and rarely is that more applicable than in the personal use of CGM and the data it provides [32]. Undoubtedly, insulin therapy would often be modified in clinical practice based on a CGM report, e. g. a premix changed for other medication and/or additional diabetes self-management education and support offered. Considering the findings of some studies, in some patients, different insulin regimens could be more effective and safer than premixed insulin in achieving the CGM-based targets [25–27]. Many people with T2D and less-intensive therapies could benefit from CGM use, with both safety and efficacy improved. We expect future research (including cost–benefit analyses) to expand the role of CGM also in premixed-insulin patients.

The standard glycemic parameters (measured HbA1c, preprandial and postprandial glucose) were all slightly above the recommended targets for the general type 2 diabetes population but satisfactory for older adults [3, 5, 13]. The patients received standard guideline-informed concomitant antihyperglycaemic therapy that was not expected to cause or contribute to hypoglycemia [2, 3, 5].

Our sample size was small due to the limitations in study staff and personal access to the outpatient clinic after COVID-19. In addition, a significant number of invitees (26%) refused to participate in the study, which could result in sampling bias and affect the generalizability of the results. We also did not include the patients with the worst glycaemic control, since a timely therapeutical intervention was considered the priority. Our study is also purely descriptive. On the other hand, we presented evidence from a real-world setting based on the best available quality CGM and laboratory results. GMI and laboratory HbA1c were identical in our patients, indicating stable glucose control over a period longer than 10 days [17, 33] and suggesting the patients continued with their usual glycaemic control routine while using a glucose sensor. TIR and laboratory HbA1c also corresponded well, since the HbA1c value of 57.4 mmol/mol (7.4%) indicates the TIR of approx. 60% in general [32, 33].

## Conclusions

Our real-world observational study in T2D patients treated with low-premixed insulin shows that most of them did not meet the recommended TBR target for older/high-risk patients while reaching the TIR and TAR targets. Nevertheless, the time spent in hypoglycemia (also nocturnal) was relatively short. The study indicates that the general T2D population targets would mostly be met for TBR and %CV in our population but not the TIR and TAR targets. In our premix-treated patients, CGM appears to be a valuable and informative clinical tool for managing and selecting antihyperglycaemic therapy.

## Data Availability

The data sets generated and analysed during the current study are not publicly available due to some patients’ objections to publicly presenting their raw data. Still, with limitations, they are available from the corresponding author upon reasonable request.
